# 2,2′-{[(2,2′-Dieth­oxy-1,1′-binaphthalene-6,6′-di­yl)bis­(4,1-phenyl­ene)]bis­(methan­ylyl­idene)}dimalononitrile

**DOI:** 10.1107/S1600536812037282

**Published:** 2012-09-05

**Authors:** Shikun Chen

**Affiliations:** a Department of Chemistry and Chemical Engineering, Huainan Normal University, 278 Xueyuan Road, Tianjiaan District, Huainan 232001, People’s Republic of China

## Abstract

The title compound, C_44_H_30_N_4_O_2_, was prepared from 6,6′-dibromo-2,2′-dieth­oxy-1,1′-binaphthalene through a coupling reaction with 4-(4,4,5,5-tetra­methyl-1,3,2-dioxaborolan-2-yl)benzaldehyde followed by a Knoevenagel reaction with malononitrile. The dihedral angle between the symmetry-related naphthalene ring systems is 68.82 (8)° while the dihedral angle between the the naphthalene ring system and the adjacent benzene ring is 16.92 (7)°. Four symmetry-independent mol­ecules which are linked by inter­molecular C—H⋯π inter­action generate the packing motif in the crystal structure. One of the CN groups is disordered over two sets of sites in a 0.60 (2):0.40 (2) ratio.

## Related literature

For applications of 6,6′-dibromo-[1,1′-binaphthalene]-2,2′-diol and its derivatives in asymmetric synthesis, see: Hu *et al.* (1996 ▶); Lou *et al.* (2006 ▶); Brunel (2006 ▶). For standard bond lengths, see: Allen *et al.* (1987 ▶). 
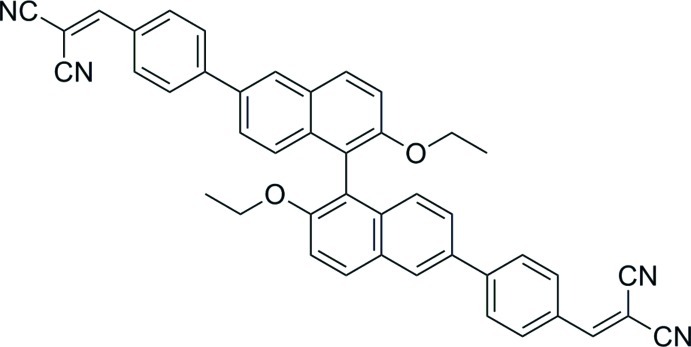


## Experimental

### 

#### Crystal data

C_44_H_30_N_4_O_2_
*M* *_r_* = 646.72
Tetragonal, 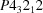

*a* = 8.4556 (12) Å
*c* = 46.991 (9) Å
*V* = 3359.7 (10) Å^3^
*Z* = 4
Mo *K*α radiation
μ = 0.08 mm^−1^
*T* = 173 K
0.24 × 0.15 × 0.08 mm

#### Data collection

Rigaku MM007HF diffractometer with Saturn724+ CCD detector
Absorption correction: multi-scan (*CrystalClear*; Rigaku/MSC, 2008 ▶) *T*_min_ = 0.789, *T*_max_ = 1.000
11840 measured reflections
1901 independent reflections
1825 reflections with *I* > 2σ(*I*)
*R*_int_ = 0.043

#### Refinement

*R*[*F* ^2^ > 2σ(*F* ^2^)] = 0.043
*wR*(*F* ^2^) = 0.095
*S* = 1.15
1901 reflections
246 parameters
40 restraints
H-atom parameters constrained
Δρ_max_ = 0.17 e Å^−3^
Δρ_min_ = −0.14 e Å^−3^

### 

Data collection: *CrystalClear* (Rigaku/MSC, 2008 ▶); cell refinement: *CrystalClear*; data reduction: *CrystalClear*; program(s) used to solve structure: *SHELXS97* (Sheldrick, 2008 ▶); program(s) used to refine structure: *SHELXL97* (Sheldrick, 2008 ▶); molecular graphics: *Mercury* (Macrae *et al.*, 2006 ▶); software used to prepare material for publication: *SHELXL97*.

## Supplementary Material

Crystal structure: contains datablock(s) global, I. DOI: 10.1107/S1600536812037282/qm2082sup1.cif

Supplementary material file. DOI: 10.1107/S1600536812037282/qm2082Isup2.mol

Structure factors: contains datablock(s) I. DOI: 10.1107/S1600536812037282/qm2082Isup3.hkl

Supplementary material file. DOI: 10.1107/S1600536812037282/qm2082Isup4.cml

Additional supplementary materials:  crystallographic information; 3D view; checkCIF report

## Figures and Tables

**Table 1:** Hydrogen-bond geometry (Å, °) *Cg*2 and *Cg*3 are the centroids of the C11-C16 and C14/C15/C17–C20 rings, respectively.

*D*—H⋯*A*	*D*—H	H⋯*A*	*D*⋯*A*	*D*—H⋯*A*
C4—H4⋯*Cg*3^i^	0.95	2.90	3.710 (3)	144
C10—H10⋯*Cg*2^i^	0.95	2.50	3.363 (3)	150
C22—H22*C*⋯*Cg*2^ii^	0.98	2.94	3.769 (3)	143

Symmetry codes: (i) 
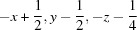
; (ii) 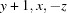.

**Fig. 1.**
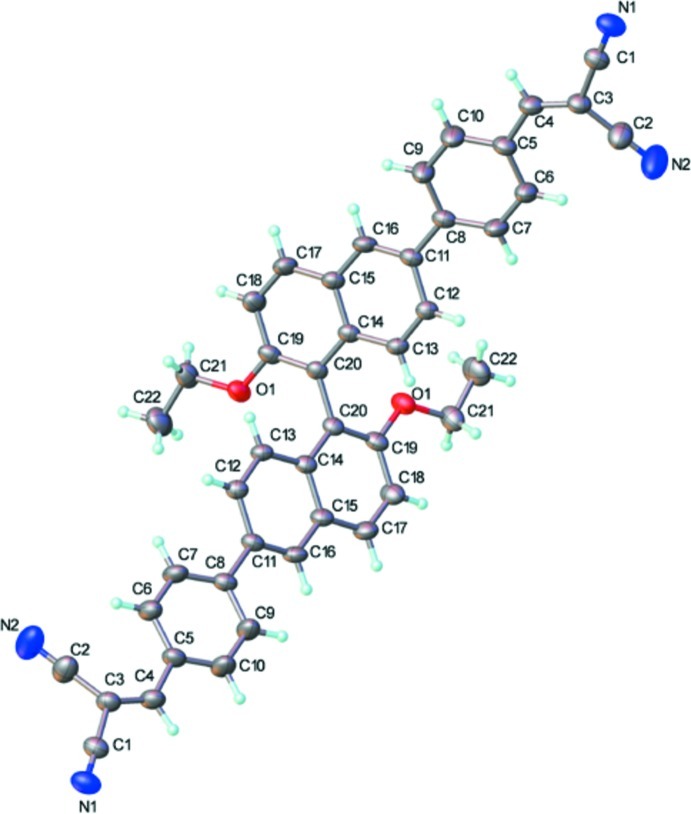
View of the title compound with 35% probability ellipsoid and the atom-numbering scheme.

**Fig. 2.**
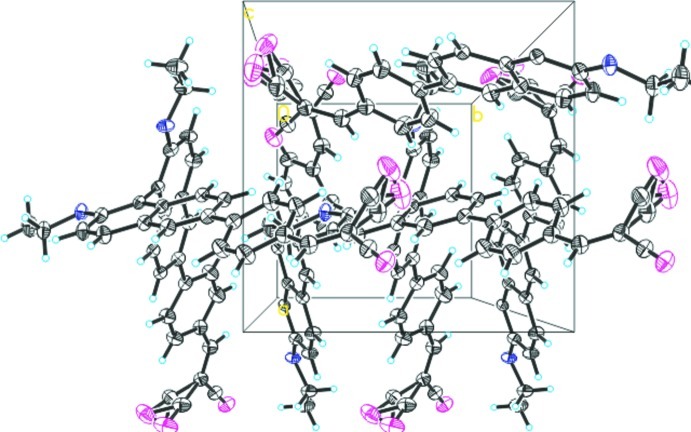
Crystal packing of the title compound [symmetry code: (i) x+1/2, -y+1/2, -z+1/4].

## supplementary crystallographic information

### Comment

Chiral compounds especially when used as chiral ligands are particularly important in asymmetric synthesis. 6,6'-dibromo-[1,1'-binaphthalene]-2,2'-diol and its derivatives have received considerable attention in the literature. They are attractive from several points of view in application (Hu *et al.*, 1996; Lou *et al.*, 2006; Brunel, 2006). As part of our search for new 6,6'-dibromo-[1,1'-binaphthalene]-2,2'-diol compounds, we synthesized the title compound (I), whose X-ray crystal structure is reported herein. No classical inter- or intramolecular hydrogen bonds were found in the structure. Bond lengths (Allen *et al.*, 1987) and angles are within normal ranges. The angle between the planes of the naphthalene rings is 68.82 °.

### Experimental

6,6'-dibromo-2,2'-diethoxy-1,1'-binaphthalene (1 g, 2 mmol) in dry THF (45 ml) was treated with 4-(4,4,5,5-tetramethyl-1,3,2-dioxaborolan-2-yl)benzaldehyde (1 g, 4.3 mmol) through a coupling reaction to give 4,4'-(2,2'-diethoxy-[1,1'-binaphthalene]-6,6'-diyl)dibenzaldehyde which then reacted with malononitrile to give the title compound as a yellow solid in 83% yield (2 steps). Single crystals of the title compound suitable for X-ray diffraction were obtained by slow evaporation of CH\2Cl\2/n-hexane solution over a period of several days.

### Refinement

All H atoms were placed in calculated positions, (C - H = 0.95 Å for aromatic, 0.99 Å for methylene and 0.98 Å for methyl H atoms), and included in the final cycles of refinement using a riding model, with *U*_iso_(H) = 1.2 *U*_eq_(C) or 1.5 *U*_eq_(C) for methyl H atoms. In the absence of significant anomalous scattering effects, Friedel pairs were merged in the final refinement.

### Crystal data

**Table:**

C_44_H_30_N_4_O_2_	*D*_x_ = 1.279 Mg m^−^^3^
*M**_r_* = 646.72	Mo *K*α radiation, λ = 0.71073 Å
Tetragonal, *P*4_3_2_1_2	Cell parameters from 7189 reflections
*a* = 8.4556 (12) Å	θ = 1.3–25.4°
*c* = 46.991 (9) Å	µ = 0.08 mm^−^^1^
*V* = 3359.7 (10) Å^3^	*T* = 173 K
*Z* = 4	Plate, yellow
*F*(000) = 1352	0.24 × 0.15 × 0.08 mm

### Data collection

**Table:**

Rigaku MM007HF diffractometer with Saturn724+ CCD detector	1901 independent reflections
Radiation source: Rotating Anode	1825 reflections with *I* > 2σ(*I*)
Confocal monochromator	*R*_int_ = 0.043
Detector resolution: 28.5714 pixels mm^-1^	θ_max_ = 25.3°, θ_min_ = 2.5°
ω scans at fixed χ = 45°	*h* = −9→9
Absorption correction: multi-scan (*CrystalClear*; Rigaku/MSC, 2008)	*k* = −8→10
*T*_min_ = 0.789, *T*_max_ = 1.000	*l* = −56→55
11840 measured reflections	

### Refinement

**Table:**

Refinement on *F*^2^	Primary atom site location: structure-invariant direct methods
Least-squares matrix: full	Secondary atom site location: difference Fourier map
*R*[*F*^2^ > 2σ(*F*^2^)] = 0.043	Hydrogen site location: inferred from neighbouring sites
*wR*(*F*^2^) = 0.095	H-atom parameters constrained
*S* = 1.15	*w* = 1/[σ^2^(*F*_o_^2^) + (0.0325*P*)^2^ + 1.0613*P*] where *P* = (*F*_o_^2^ + 2*F*_c_^2^)/3
1901 reflections	(Δ/σ)_max_ = 0.001
246 parameters	Δρ_max_ = 0.17 e Å^−^^3^
40 restraints	Δρ_min_ = −0.14 e Å^−^^3^

### Special details

**Table:**

Geometry. All e.s.d.'s (except the e.s.d. in the dihedral angle between two l.s. planes) are estimated using the full covariance matrix. The cell e.s.d.'s are taken into account individually in the estimation of e.s.d.'s in distances, angles and torsion angles; correlations between e.s.d.'s in cell parameters are only used when they are defined by crystal symmetry. An approximate (isotropic) treatment of cell e.s.d.'s is used for estimating e.s.d.'s involving l.s. planes.
Refinement. Refinement of *F*^2^ against ALL reflections. The weighted *R*-factor *wR* and goodness of fit *S* are based on *F*^2^, conventional *R*-factors *R* are based on *F*, with *F* set to zero for negative *F*^2^. The threshold expression of *F*^2^ > σ(*F*^2^) is used only for calculating *R*-factors(gt) *etc*. and is not relevant to the choice of reflections for refinement. *R*-factors based on *F*^2^ are statistically about twice as large as those based on *F*, and *R*- factors based on ALL data will be even larger.

### Fractional atomic coordinates and isotropic or equivalent isotropic displacement parameters (Å^2^)

**Table:**

	*x*	*y*	*z*	*U*_iso_*/*U*_eq_	Occ. (<1)
O1	0.7522 (2)	0.4017 (2)	0.00856 (3)	0.0384 (4)	
N1	−0.4620 (3)	0.2058 (3)	−0.23834 (4)	0.0487 (6)	
N2	−0.4836 (17)	0.5495 (19)	−0.1722 (4)	0.084 (5)	0.40 (2)
C2	−0.4126 (17)	0.4452 (17)	−0.1812 (3)	0.053 (3)	0.40 (2)
N2'	−0.5300 (8)	0.4570 (17)	−0.15877 (19)	0.083 (3)	0.60 (2)
C2'	−0.4429 (10)	0.3911 (14)	−0.17341 (18)	0.052 (2)	0.60 (2)
C1	−0.4034 (3)	0.2527 (3)	−0.21824 (5)	0.0393 (6)	
C3	−0.3335 (3)	0.3113 (3)	−0.19219 (4)	0.0378 (6)	
C4	−0.1873 (3)	0.2674 (3)	−0.18449 (5)	0.0416 (7)	
H4	−0.1352	0.2004	−0.1977	0.050*	
C5	−0.0950 (3)	0.3054 (3)	−0.15924 (4)	0.0382 (6)	
C6	−0.1387 (3)	0.4106 (3)	−0.13769 (5)	0.0433 (7)	
H6	−0.2366	0.4654	−0.1388	0.052*	
C7	−0.0394 (3)	0.4350 (3)	−0.11469 (4)	0.0429 (7)	
H7	−0.0706	0.5077	−0.1003	0.051*	
C8	0.1050 (3)	0.3565 (3)	−0.11186 (4)	0.0343 (6)	
C9	0.1473 (3)	0.2537 (3)	−0.13358 (5)	0.0465 (7)	
H9	0.2446	0.1981	−0.1324	0.056*	
C10	0.0515 (4)	0.2308 (4)	−0.15674 (5)	0.0510 (8)	
H10	0.0859	0.1623	−0.1715	0.061*	
C11	0.2097 (3)	0.3794 (3)	−0.08678 (4)	0.0323 (6)	
C12	0.1510 (3)	0.4466 (3)	−0.06116 (4)	0.0317 (6)	
H12	0.0432	0.4778	−0.0602	0.038*	
C13	0.2448 (3)	0.4678 (3)	−0.03786 (4)	0.0300 (5)	
H13	0.2009	0.5144	−0.0212	0.036*	
C14	0.4059 (3)	0.4221 (3)	−0.03779 (4)	0.0298 (5)	
C15	0.4660 (3)	0.3549 (3)	−0.06345 (4)	0.0330 (6)	
C16	0.3656 (3)	0.3356 (3)	−0.08715 (4)	0.0373 (6)	
H16	0.4077	0.2905	−0.1040	0.045*	
C17	0.6258 (3)	0.3076 (3)	−0.06415 (5)	0.0407 (7)	
H17	0.6681	0.2644	−0.0812	0.049*	
C18	0.7207 (3)	0.3226 (3)	−0.04090 (4)	0.0402 (6)	
H18	0.8278	0.2891	−0.0418	0.048*	
C19	0.6604 (3)	0.3879 (3)	−0.01535 (4)	0.0336 (6)	
C20	0.5060 (3)	0.4402 (3)	−0.01356 (4)	0.0290 (5)	
C21	0.9066 (3)	0.3277 (4)	0.00773 (5)	0.0444 (7)	
H21B	0.9728	0.3782	−0.0071	0.053*	
H21A	0.8958	0.2140	0.0031	0.053*	
C22	0.9817 (4)	0.3469 (4)	0.03612 (5)	0.0612 (9)	
H22C	1.0860	0.2964	0.0360	0.092*	
H22A	0.9151	0.2971	0.0507	0.092*	
H22B	0.9935	0.4597	0.0404	0.092*	

### Atomic displacement parameters (Å^2^)

**Table:**

	*U*^11^	*U*^22^	*U*^33^	*U*^12^	*U*^13^	*U*^23^
O1	0.0297 (10)	0.0549 (12)	0.0307 (8)	−0.0026 (9)	−0.0028 (7)	0.0034 (8)
N1	0.0508 (15)	0.0606 (16)	0.0348 (10)	−0.0123 (13)	−0.0046 (11)	−0.0004 (11)
N2	0.074 (7)	0.088 (8)	0.089 (8)	0.031 (6)	−0.031 (6)	−0.036 (7)
C2	0.049 (6)	0.055 (6)	0.056 (6)	0.006 (5)	−0.013 (4)	−0.017 (5)
N2'	0.059 (4)	0.127 (8)	0.062 (4)	0.017 (4)	−0.008 (3)	−0.035 (5)
C2'	0.043 (4)	0.075 (5)	0.039 (3)	−0.001 (4)	−0.005 (3)	−0.010 (3)
C1	0.0405 (16)	0.0455 (16)	0.0320 (11)	−0.0067 (13)	−0.0014 (11)	0.0021 (11)
C3	0.0402 (16)	0.0434 (16)	0.0298 (11)	−0.0030 (13)	−0.0034 (11)	−0.0053 (11)
C4	0.0444 (17)	0.0506 (17)	0.0299 (11)	0.0004 (14)	−0.0020 (11)	−0.0085 (12)
C5	0.0461 (16)	0.0423 (16)	0.0261 (10)	−0.0009 (13)	−0.0048 (11)	−0.0045 (10)
C6	0.0406 (16)	0.0567 (18)	0.0326 (12)	0.0080 (14)	−0.0031 (11)	−0.0093 (12)
C7	0.0448 (16)	0.0551 (18)	0.0287 (11)	0.0052 (14)	0.0002 (11)	−0.0127 (11)
C8	0.0410 (15)	0.0383 (15)	0.0236 (10)	−0.0049 (12)	0.0002 (10)	−0.0006 (10)
C9	0.0489 (17)	0.0541 (18)	0.0366 (12)	0.0131 (15)	−0.0110 (12)	−0.0146 (12)
C10	0.0564 (19)	0.0589 (19)	0.0378 (13)	0.0142 (16)	−0.0111 (13)	−0.0197 (13)
C11	0.0376 (15)	0.0367 (14)	0.0226 (10)	−0.0047 (12)	−0.0007 (10)	0.0004 (10)
C12	0.0319 (14)	0.0358 (14)	0.0272 (10)	−0.0034 (11)	0.0015 (9)	−0.0020 (10)
C13	0.0337 (14)	0.0337 (13)	0.0227 (10)	−0.0034 (11)	0.0040 (9)	−0.0031 (9)
C14	0.0328 (14)	0.0331 (14)	0.0236 (10)	−0.0053 (11)	0.0031 (9)	0.0022 (9)
C15	0.0363 (15)	0.0392 (15)	0.0236 (10)	−0.0031 (11)	0.0047 (10)	0.0010 (10)
C16	0.0437 (16)	0.0470 (16)	0.0213 (10)	−0.0006 (13)	0.0048 (10)	−0.0040 (10)
C17	0.0400 (16)	0.0538 (18)	0.0282 (11)	0.0033 (13)	0.0077 (11)	−0.0033 (12)
C18	0.0308 (14)	0.0561 (18)	0.0338 (11)	−0.0001 (13)	0.0056 (11)	0.0038 (12)
C19	0.0354 (15)	0.0413 (15)	0.0242 (10)	−0.0066 (12)	0.0007 (10)	0.0056 (10)
C20	0.0303 (14)	0.0321 (14)	0.0245 (10)	−0.0042 (11)	0.0033 (9)	0.0045 (10)
C21	0.0308 (15)	0.0590 (19)	0.0433 (13)	−0.0011 (14)	−0.0002 (11)	0.0104 (13)
C22	0.0456 (19)	0.084 (3)	0.0536 (15)	0.0034 (17)	−0.0156 (14)	0.0032 (17)

### Geometric parameters (Å, º)

**Table:**

O1—C19	1.371 (3)	C11—C12	1.421 (3)
O1—C21	1.448 (3)	C12—C13	1.364 (3)
N1—C1	1.138 (3)	C12—H12	0.9500
N2—C2	1.148 (8)	C13—C14	1.416 (3)
C2—C3	1.413 (9)	C13—H13	0.9500
N2'—C2'	1.151 (6)	C14—C20	1.427 (3)
C2'—C3	1.446 (7)	C14—C15	1.426 (3)
C1—C3	1.447 (3)	C15—C16	1.410 (3)
C3—C4	1.340 (4)	C15—C17	1.410 (4)
C4—C5	1.456 (3)	C16—H16	0.9500
C4—H4	0.9500	C17—C18	1.361 (3)
C5—C10	1.395 (4)	C17—H17	0.9500
C5—C6	1.398 (3)	C18—C19	1.416 (3)
C6—C7	1.384 (3)	C18—H18	0.9500
C6—H6	0.9500	C19—C20	1.381 (3)
C7—C8	1.397 (4)	C20—C20^i^	1.498 (4)
C7—H7	0.9500	C21—C22	1.487 (3)
C8—C9	1.387 (3)	C21—H21B	0.9900
C8—C11	1.486 (3)	C21—H21A	0.9900
C9—C10	1.370 (3)	C22—H22C	0.9800
C9—H9	0.9500	C22—H22A	0.9800
C10—H10	0.9500	C22—H22B	0.9800
C11—C16	1.369 (4)		
			
C19—O1—C21	116.82 (19)	C12—C13—C14	121.7 (2)
N2—C2—C3	176.6 (14)	C12—C13—H13	119.1
N2'—C2'—C3	178.8 (9)	C14—C13—H13	119.1
N1—C1—C3	178.2 (3)	C13—C14—C20	122.9 (2)
C4—C3—C2	124.0 (5)	C13—C14—C15	116.7 (2)
C4—C3—C2'	123.7 (4)	C20—C14—C15	120.4 (2)
C2—C3—C2'	25.8 (5)	C16—C15—C17	121.7 (2)
C4—C3—C1	120.7 (2)	C16—C15—C14	120.0 (2)
C2—C3—C1	113.0 (6)	C17—C15—C14	118.3 (2)
C2'—C3—C1	114.5 (4)	C11—C16—C15	122.6 (2)
C3—C4—C5	130.8 (2)	C11—C16—H16	118.7
C3—C4—H4	114.6	C15—C16—H16	118.7
C5—C4—H4	114.6	C18—C17—C15	121.3 (2)
C10—C5—C6	117.5 (2)	C18—C17—H17	119.3
C10—C5—C4	116.4 (2)	C15—C17—H17	119.3
C6—C5—C4	126.1 (2)	C17—C18—C19	120.3 (2)
C7—C6—C5	120.0 (2)	C17—C18—H18	119.8
C7—C6—H6	120.0	C19—C18—H18	119.8
C5—C6—H6	120.0	O1—C19—C20	117.2 (2)
C6—C7—C8	122.3 (2)	O1—C19—C18	121.6 (2)
C6—C7—H7	118.9	C20—C19—C18	121.1 (2)
C8—C7—H7	118.9	C19—C20—C14	118.5 (2)
C9—C8—C7	116.9 (2)	C19—C20—C20^i^	121.5 (2)
C9—C8—C11	120.8 (2)	C14—C20—C20^i^	119.9 (2)
C7—C8—C11	122.3 (2)	O1—C21—C22	108.3 (2)
C10—C9—C8	121.4 (3)	O1—C21—H21B	110.0
C10—C9—H9	119.3	C22—C21—H21B	110.0
C8—C9—H9	119.3	O1—C21—H21A	110.0
C9—C10—C5	121.9 (2)	C22—C21—H21A	110.0
C9—C10—H10	119.1	H21B—C21—H21A	108.4
C5—C10—H10	119.1	C21—C22—H22C	109.5
C16—C11—C12	117.1 (2)	C21—C22—H22A	109.5
C16—C11—C8	121.9 (2)	H22C—C22—H22A	109.5
C12—C11—C8	121.1 (2)	C21—C22—H22B	109.5
C13—C12—C11	122.0 (2)	H22C—C22—H22B	109.5
C13—C12—H12	119.0	H22A—C22—H22B	109.5
C11—C12—H12	119.0		
			
N2—C2—C3—C4	−137 (20)	C8—C11—C12—C13	179.6 (2)
N2—C2—C3—C2'	−39 (19)	C11—C12—C13—C14	−0.8 (4)
N2—C2—C3—C1	60 (21)	C12—C13—C14—C20	−178.8 (2)
N2'—C2'—C3—C4	117 (46)	C12—C13—C14—C15	0.9 (3)
N2'—C2'—C3—C2	18 (45)	C13—C14—C15—C16	−0.5 (3)
N2'—C2'—C3—C1	−75 (46)	C20—C14—C15—C16	179.2 (2)
N1—C1—C3—C4	134 (10)	C13—C14—C15—C17	−179.8 (2)
N1—C1—C3—C2	−62 (10)	C20—C14—C15—C17	−0.1 (4)
N1—C1—C3—C2'	−34 (10)	C12—C11—C16—C15	0.1 (4)
C2—C3—C4—C5	20.8 (11)	C8—C11—C16—C15	−179.3 (2)
C2'—C3—C4—C5	−10.3 (8)	C17—C15—C16—C11	179.3 (2)
C1—C3—C4—C5	−177.6 (3)	C14—C15—C16—C11	0.1 (4)
C3—C4—C5—C10	175.7 (3)	C16—C15—C17—C18	−178.1 (3)
C3—C4—C5—C6	−5.3 (5)	C14—C15—C17—C18	1.1 (4)
C10—C5—C6—C7	−1.4 (4)	C15—C17—C18—C19	−0.5 (4)
C4—C5—C6—C7	179.7 (3)	C21—O1—C19—C20	172.0 (2)
C5—C6—C7—C8	−0.5 (4)	C21—O1—C19—C18	−8.0 (3)
C6—C7—C8—C9	1.2 (4)	C17—C18—C19—O1	178.8 (2)
C6—C7—C8—C11	−178.3 (2)	C17—C18—C19—C20	−1.2 (4)
C7—C8—C9—C10	0.1 (4)	O1—C19—C20—C14	−177.8 (2)
C11—C8—C9—C10	179.6 (3)	C18—C19—C20—C14	2.2 (4)
C8—C9—C10—C5	−2.1 (5)	O1—C19—C20—C20^i^	−1.4 (3)
C6—C5—C10—C9	2.7 (4)	C18—C19—C20—C20^i^	178.6 (2)
C4—C5—C10—C9	−178.3 (3)	C13—C14—C20—C19	178.2 (2)
C9—C8—C11—C16	17.4 (4)	C15—C14—C20—C19	−1.5 (3)
C7—C8—C11—C16	−163.2 (3)	C13—C14—C20—C20^i^	1.7 (3)
C9—C8—C11—C12	−162.0 (2)	C15—C14—C20—C20^i^	−177.98 (19)
C7—C8—C11—C12	17.5 (4)	C19—O1—C21—C22	−176.2 (2)
C16—C11—C12—C13	0.3 (4)		

Symmetry code: (i) *y*, *x*, −*z*.

### Hydrogen-bond geometry (Å, º)

Cg2 and Cg3 are the centroids of the C11-C16 and C14/C15/C17–C20 rings, respectively.

**Table:**

*D*—H···*A*	*D*—H	H···*A*	*D*···*A*	*D*—H···*A*
C4—H4···*Cg*3^ii^	0.95	2.90	3.710 (3)	144
C10—H10···*Cg*2^ii^	0.95	2.50	3.363 (3)	150
C22—H22*C*···*Cg*2^iii^	0.98	2.94	3.769 (3)	143

Symmetry codes: (ii) −*x*+1/2, *y*−1/2, −*z*−1/4; (iii) *y*+1, *x*, −*z*.
